# Application and Uses of Electronic Noses for Clinical Diagnosis on Urine Samples: A Review

**DOI:** 10.3390/s16101708

**Published:** 2016-10-14

**Authors:** Laura Capelli, Gianluigi Taverna, Alessia Bellini, Lidia Eusebio, Niccolò Buffi, Massimo Lazzeri, Giorgio Guazzoni, Giorgio Bozzini, Mauro Seveso, Alberto Mandressi, Lorenzo Tidu, Fabio Grizzi, Paolo Sardella, Giuseppe Latorre, Rodolfo Hurle, Giovanni Lughezzani, Paolo Casale, Sara Meregali, Selena Sironi

**Affiliations:** 1 Politecnico di Milano, Dipartimento di Chimica, Materiali e Ingegneria Chimica “Giulio Natta”, piazza Leonardo da Vinci 32, Milan 20133, Italy; alessia1.bellini@mail.polimi.it (A.B.); lidia.eusebio@polimi.it (L.E.); selena.sironi@polimi.it (S.S.); 2 Department of Urology, Humanitas Clinical and Research Center, Via Manzoni 56, Rozzano, Milan 20089, Italy; gianluigi.taverna@humanitas.it (G.T.); niccolo.buffi@hunimed.it (N.B.); massimo.lazzeri@humanitas.it (M.L.); giorgio.guazzoni@humanitas.it (G.G.); Rodolfo.hurle@humanitas.it (R.H.); giovanni.lughezzani@humanitas.it (G.L.); paolo.casale@humanitas.it (P.C.); 3 Ospedale Humanitas Mater Domini, Via Gerenzano 2, Castellanza, Varese 21053, Italy; giorgio.bozzini@HMD.it (G.B.); mauro.seveso@humanitas.it (M.S.); alberto.mandressi@auro.it (A.M.); sara.meregali@HMD.it (S.M.); 4 Italian Ministry of Defense’s, Military Veterinary Center, CEMIVET, Via Provinciale Castiglionese, 201, Grosseto 58100, Italy; lorudit@virgilio.it (L.T.); francescopaolo.sardella@esercito.difesa.it (P.S.); siegher@gmail.com (G.L.); 5 Department of Immunology and Inflammation, Humanitas Clinical and Research Center, Via Manzoni 56, Rozzano, Milan 20089, Italy; fabio.grizzi@humanitasreserch.it

**Keywords:** urine analysis, olfaction, prostate cancer, VOCs, biomarkers, J0101

## Abstract

The electronic nose is able to provide useful information through the analysis of the volatile organic compounds in body fluids, such as exhaled breath, urine and blood. This paper focuses on the review of electronic nose studies and applications in the specific field of medical diagnostics based on the analysis of the gaseous headspace of human urine, in order to provide a broad overview of the state of the art and thus enhance future developments in this field. The research in this field is rather recent and still in progress, and there are several aspects that need to be investigated more into depth, not only to develop and improve specific electronic noses for different diseases, but also with the aim to discover and analyse the connections between specific diseases and the body fluids odour. Further research is needed to improve the results obtained up to now; the development of new sensors and data processing methods should lead to greater diagnostic accuracy thus making the electronic nose an effective tool for early detection of different kinds of diseases, ranging from infections to tumours or exposure to toxic agents.

## 1. Introduction

Urine odour analysis by sensorial techniques, i.e., smelling, is maybe one of the most antique methods for the detection of specific pathologies. As a matter of fact, several pathological processes, such as infection and endogenous metabolic disorders, can affect human body odours [1]. Already in 400 BC Hippocrates recognized the diagnostic usefulness of body odours, reporting different disease-specific odours emanated from urine [2].

Urine is a very complex organic fluid from the chemical point of view, due to its high number of constituents [3,4,5], and to its extremely variable composition; it is proved that urine composition can vary depending on a multitude of factors, including gender, age, hormonal status, food habits, physical activity, and presence of specific pathologies [6,7,8,9].

In the scientific literature there are some studies regarding the evaluation of urine composition by means of chemical analyses applied either to liquid urine [10,11] or to its gaseous headspace [3,12], which highlight this complexity. Furthermore, the sampling method is critical, as it may alter the urine profile [3,13,14]. Despite these difficulties, there is a lot of recent research about the identification of biomarkers in urine for early and non-invasive diagnosis purposes (e.g., [15,16,17]).

However, in all those cases in which the characterization of urine odour is preferred over a detailed chemical mapping, sensorial analysis can be successfully applied. In general, odour analysis is not simple, as it entails the objectification of a sensation; however, in the last decades, specific techniques for odour characterization and measurement have been implemented and developed, especially for the application to the environmental sector [18,19].

Sensorial techniques are based on the principle of characterizing odours referring to the sensation caused by an odorous sample directly on a panel of human assessors. Among these, the most widespread technique is dynamic olfactometry [20], which is now widely applied for testing odours for environmental management purposes [21,22]. Dynamic olfactometry determines the odour concentration of an “odorous air sample” relating to the sensation caused by the sample directly on a panel of opportunely selected people. Odour concentration is expressed in European odour units per cubic meter (ou_E_/m^3^), and it represents the number of dilutions with neutral air required to bring the sample to its odour detection threshold concentration [23].

Even though chemical analysis is a more consolidated method, it can turn out to be highly complex and not always effective for odour analysis. This is particularly true in the characterization of complex odours, for which it is difficult to relate the sensation provoked by an odorous mixture in humans to its chemical composition [18], mostly due to the highly complicated effects of odorant mixing [24,25]. There are also other sensorial methods, which allow odour hedonic tone [26], intensity [27] and quality [28,29] to be evaluated, as well.

Referring to urine odour analysis, some examples of the application of such sensorial techniques exist, regarding for instance the evaluation of odour release from sanitary pads for urinary incontinence [30]. However, for this purpose, in order to overcome mainly problems related to the panel safety, the use of purposely formulated synthetic urine was preferred over real urine samples.

In the field of medical diagnosis, there are several studies regarding the identification of specific biomarkers [3,4,5,12]; however, the most promising results published up to now were obtained relying on the sense of smell of specifically trained dogs [31,32,33]. Recently, Taverna et al. [34] reported the capability of two highly trained dogs to discriminate urine samples of patients having prostate cancer from healthy ones, achieving a diagnostic accuracy in terms of both sensitivity and specificity of over 97%, and subsequently for detecting biochemical recurrence following radical prostatectomy [35].

As previously mentioned, sensorial techniques involving the use of animals or humans, when applied to the direct analysis of real urine samples, may pose some problems. Among them, their reproducibility and practical applicability (e.g., the training approach standardization of the dog), which makes them hardly suitable for the development of large-scale diagnostics.

For these reasons, the possibility of developing an instrumental method capable of reproducing the activity of the mammalian sense of smell appears as a very interesting challenge for the definition of a modern strategy for early and non-invasive diagnosis of different diseases based on the analysis of urine odour.

Electronic noses, being defined as instruments that try to emulate the human olfactory system [36,37], could be used for this purpose. Since the design of an electronic nose using chemical sensors and pattern recognition was first reported by Persaud and Dodd [38], electronic noses and their applications have been studied by several researchers throughout the world. Different studies concern the application of electronic noses in the food industry [39,40], and in recent years also developments in the environmental sector have become an issue of increasing interest [41].

One particularly interesting field of application for this technology is medical diagnostics. Even though for this extremely delicate purpose, particular attention should be given to the electronic noses limitations and all the critical aspects associated with their use [42]. Here, we decided review electronic nose studies and applications in the specific field of medical diagnosis based on the analysis of urine odour. Despite the variety of works regarding more generically biomedical applications of electronic noses [43,44], the scientific literature regarding specifically their utilization for the analysis of urine samples is not so huge, for this reason it was possible to make an exhaustive inspection among all the research papers regarding this particular issue, considering a timeframe of publication between 1997 and 2015. The aim of this paper is therefore to provide a broad overview of the state of the art and thus enhance future developments in this specific field.

It is not the aim of this paper to focus on the details of electronic nose technology, for this reason no specific indications about the electronic nose design and operation are given here, thereby we remand readers to other exhaustive review papers or handbooks on this subject [39,43,45,46].

## 2. Electronic Noses for Urine Sample Analysis

### 2.1. General Overview

Recently, several researches have been published regarding the investigation of specific Volatile Organic Compounds (VOCs) in urine, in order to determine their origin and/or the reasons for their presence, giving that different devices have been developed for the purpose of VOCs detection and identification (e.g., Proton Transfer Reaction Mass Spectrometry (PTR-MS), gas chromatography-mass spectrometry (GC-MS) [3,8,9]). The development of these techniques of course applies not only to urine analysis but also to other fields, such as, for example, food analysis, cosmetics or biomedicine.

In the latter, one main target is evaluating the possibility to apply such new techniques in the field of medical diagnosis, due to the interest towards effective diagnosis by means of less-invasive and cheaper techniques.

The electronic nose might represent a promising candidate for medical diagnosis, being a technology that aims to reproduce the human sense of smell for the detection of volatile components. In the case of medical diagnosis, some volatile biomarkers can be associated with the presence of foreign bodies, such as bacteria, or to the presence of microorganisms, which is always linked to modifications of the VOCs composition. Electronic noses employ an array of typically non-specific sensors, which interact differently with VOCs: every VOC generates a characteristic fingerprint due to the interaction with the sensor array, which can then be analysed by means of an appropriate pattern recognition system, in order to investigate its nature and origin. In general, an electronic nose device can be assumed to be composed of three different parts: a sample delivery system, a detection system and a data computing system [47].

The sample delivery system introduces odour samples into the detection system: it can include a pre-treatment step in order to enrich the percentage of VOCs and improve the quality of the detection. The second part, i.e., the detection system, consists of sets of gas sensors which interact directly with the odours to be analysed: the most commonly used are metal-oxide sensors, polymeric sensors and quartz microbalance sensors, each type providing a different answer to a different component part of the body fluid analysed. Data are processed exploiting different techniques of multivariate statistical analysis, the most common being Principal Component Analysis (PCA) and Linear Discriminant Analysis (LDA) [48], which usually provide the best performance in this field. PCA is a linear feature extraction technique which focuses on visualizing data within a low dimensional space, thereby maintaining the main information contained in the original data. LDA provides an algorithm to find a linear combination of features that characterizes or separates two or more classes of objects or events, aiming to achieve maximum separation between data classes and dimensionality reduction before later classification. However, it is necessary to take into account that many other methods for data processing are still in the experimental stage, often with remarkable results.

This review aims to describe the use of electronic noses in the biomedical field, focusing on different diseases that have been studied by means of the analysis of urine samples until now, and the results that have been achieved over the years, in order to give to the reader an extensive overview of the state-of the-art of this particular application.

All studies reported in literature use electronic noses for the analysis of the gaseous headspace of urine. An overview of these studies is summarized in Table 1, which, besides authors and year, reports the investigated diseases, the adopted detection system (i.e., sensor type), and the data processing methods used.

The following sections provide an overview of the state-of-the-art of the application of electronic noses for the analysis of urine samples by classifying those applications depending on the target investigated, thus including the discrimination of bacteria cultures (Section 2.2) [49,50,51,52], or the detection of urinary tract infections (Section 2.3) [53,54,55,56,57,58], cancer diseases (Section 2.4) [59,60,61,62,63,64,65,66], diabetes (Section 2.5) [67,68], kidney diseases (Section 2.6) [69,74], bowel diseases (Section 2.7) [70,71,72] and exposure to toxic agents (Section 2.8) [73] (reference [74] wasn’t included in Table 1 since it refers to breath analysis).

### 2.2. Bacterial Cultures

Electronic noses have been used for the evaluation of sample headspace or the liquid directly with different purposes. Others have used the electronic nose for the discrimination between bacteria and/or the discrimination of the production of VOCs of different bacterial strains into the sterile field. The use of electronic noses on bacterial cultures produced significant answers about its possible use as a diagnostic method: alongside promising results, it has highlighted some critical aspects and therefore it was possible to take prompt action to make the necessary improvements to the instrument. Proofs in vitro, indeed, are an important part of the studies made with electronic noses, as they gave the basis for additional trials on sample of human excreta.

The first report about use of an electronic nose for the recognition of VOCs was completed by Pavlou et al. [49]; they used an electronic nose system to find out the differences between anaerobic bacteria such as *Clostridium* and *Bacteroides fragilis*, grown on blood agar. The device was composed of 14 conducting polymer sensors and data collected from the sample gaseous headspace were analysed using PCA. The results showed that clear discrimination could be obtained between the two bacteria types with 94% of the data accounted for and no problems were observed during the trial.

While in the first experiment with the electronic nose no particular problems were noticed, in other tests researchers did not achieve the excellent results of the first one, although the conclusions of subsequent experiments were not less encouraging. Bruins et al. [50] tested a new device called “Mononose” (custom-designed electronic nose device manufactured by C-it, Zutphen, The Netherlands). It consists of a handmade single metal-oxide type semiconductor gas sensor and was used to study bacterial VOCs in the gaseous headspace produced by the liquid of culture samples; data were processed using Sliding Window-Minimum Variance matching adaptation of the Dynamic Time Warping algorithm. The experiment was conducted using 30 Mononose instruments in order to verify signal repeatability on different devices.

More in detail, the author evaluated different clinically relevant bacterial species (Table 2). For each of the species, two to eight different strains were used to take intra-species biodiversity into account. A total of 52 different strains were measured in an incubator. In order to avoid the humidity influences on the semiconductor response values, all devices were operated in an incubator at a constant ambient temperature of 37 °C in order to keep the relative humidity in the headspace constant during all experiments. Moreover, the authors optimized the culture broths mixture in order to maximize the discrimination abilities.

The results show that the diagnostic specificities varied from 100% for *Clostridium difficile* to 67% for *Enterobacter cloacae*. The average identification rate was equal to 87%. The authors obtained good results if compared with currently used identification systems (e.g., the bioMerieux API-50 test), which achieve scores of <90%. The authors also highlighted the innovative aspect of parallel testing of independent instruments, thus studying the crucial aspect of applicability and reproducibility when using a larger series of independent devices. Finally, the researchers highlighted the requirement of other proofs with biological secretions.

Urine samples inoculated with bacteria cultures have produced important information concerning the research of data analysis obtained with gas sensors. Yates et al. [51] demonstrated the applicability of chemical headspace analysis to the problem of classifying bacteria presence in blood and urine samples, inoculated with bacteria cultures. They used an Agilent 4440 mass spectrometer (Agilent Technologies Inc., Santa Clara, CA, USA), together with a 32 carbon black polymer sensor array (C320, Cyrano Sciences, Smiths Detection, Bushey, Hertfordshire, UK) that showed a change in its electrical conductivity when interacting with different VOCs. They tested the gaseous headspace of samples and data processing was performed using a variety of data reduction and pattern recognition techniques in an attempt to optimise the classification process. During the experiment, some problems were observed: in urine samples, there was a sort of “chemical noise”, connected to the composition of the urine sample itself, which influenced the bacterial classification and that was not removable. Moreover, the range of the indicators used to identify the bacteria usually connected to urinary tract infections (UTI) created some issues due to the presence of acids in the samples, which ‘poison’ the gas sensor. In blood samples, data processing was successfully performed by removing the measurement of the first two samples due to instability in the measurement. The best performances were obtained, in the case of urine samples, with non-linear models for which the highest accuracy achieved is 80%, whereas for blood samples the best results were achieved by using the Sammon map and training a standard RBF network, thereby obtaining an excellent accuracy of 100% successful classifications. The obtained results are promising, pointing out that the dataset dimensional reduction can improve the robustness of a model, and, when applied to electronic nose systems, it may improve the technology and its success rates.

Aathithan et al. [52] investigated the use of Osmetech Microbial Analyzer (OMA, Osmetech plc, Crewe, UK) for the analysis of infected and uninfected human urine: this type of electronic nose is an automated headspace analyser fitted with a detector system consisting of an array of four conducting polymer sensors, each of which responds to different volatile organic compounds, depending on its size, shape and functional groups. Bacteria strains, mainly connected to urinary tract infections, were cultured in laboratory and then inoculated into urine samples with other chemicals and reagents. Data were processed by PCA for the first preliminary study and subsequent calibration of sensors: infected samples were classified by comparison with thresholds set on the data obtained by previous test on negative-control samples, so that samples outside ranges were classified as positive. The OMA was found to have a sensitivity of 84% and a specificity of 88%. When the tolerance limit is reduced, both sensitivity and specificity decreased. The presence of false negative results is certainly an important factor to be considered. A very interesting fact is that researchers were unable to associate them to a single cause, but they had to take into account various factors, such as the presence of other volatile components or bacterial volatile products in the experiment, or, conversely, the lack of production of VOCs by the bacteria analysed. For the purpose of trying to associate the electronic nose responses with the presence of specific VOCs, the author reports some studies retrieved in the scientific literature conducted by means of GCL chromatography, reporting the VOCs produced by some bacterial species, which are ethanol, DMS, TMA and methyl mercaptan. In conclusion, instead of adding everything to promising results, it is necessary to consider the analysis of the incorrect tests, which provided useful information for improvements in subsequent experiments. Focusing the attention on the last two experiments, it is possible to make some considerations about the analyses and the information coming from bacterial cultures: trials made on these are connected to an ideal condition of the sample itself, due to a lack of external disturbances that can occur during the test and can’t be avoided when using real body fluid samples. In facts, even if they can product failures, experiments in situ gave a very important additional level of information, which can’t be ignored in diseases diagnosis.

The above mentioned studies deal with the analysis of the VOCs produced by bacteria cultures in different ways. The preparation methods are different, i.e., different bacteria families, culture broths, temperatures, and incubation and manipulation times. Also the data processing was faced in different manners. Some studies used simpler models, such as PCA [49,52], whereas other studies employed more complex models, such as Sliding Window-Minimum Variance matching adaptation of the Dynamic Time Warping algorithm [50], or models ARX, RBF and non-linear model (NN) [51]. In order to allow a more immediate comparison of the different experimental conditions, Table 2 reports a scheme of the electronic noses, bacteria types, culture broths employed in the different studies, together with a synthesis of the results obtained in function of the data processing method adopted.

The reported response accuracies obtained by the combination of the different devices and data processing vary from 65% to 100%, and the variability of the response may be connected to different factors, such as the signal acquisition, the choice of the features to be analyzed, and the culture media. Without a data optimization process the obtained results may diverge even by using the same model, as reported by Yates et al. [51] or Aathithan et al. [52]: the first tried to optimize his data set, whereas the second worked on the optimization of the “threshold” set.

It is also interesting to observe how different culture broths produce different responses. The research group of Yates et al. [51] obtained different results if analyzing blood or urine. With blood, an accuracy of 100% was achieved, whereas with urine, which is extremely variable, the maximum accuracy achieved decreases to 80%. Such differences were observed also by Bruins et al. [50] who worked on the set up of a culture broth that optimized the results.

Finally, a common point that emerges from the various studies is that sample preparation is of fundamental importance in order to get good signals and thus maximize discrimination and classification.

### 2.3. Urinary Tract Infections (UTI)

One of the areas where appropriate and effective therapy is most dependent on early diagnosis is that of urogenital infections. Urinary tract infections are a significant cause of morbidity, with a continuously rising number of patients. It was estimated that 80% of the cases are caused by *Escherichia coli* and the remaining 20% by enteric pathogens such as *Enterocci*, *Klebsiella*, *Proteus*, and fungal pathogens such as *Candida albicans*. The analysis of the VOCs connected to these diseases by means of electronic noses was investigated as a possible instrument of diagnosis.

The trials in this field reported in the following part of this section were carried out in situ: samples were collected directly from patients suffering from the investigated diseases and not inoculated with bacteria as in the previous experiments discussed. These tests are a consequent step of proofs in vitro, because in this case the conditions in which they were made are much closer to the actual conditions, i.e., the analysis of samples from patients for the diagnosis of diseases.

Researchers also studied and developed different data processing methods in order to improve the quality of classification. As a matter of fact, a better analysis of data collected from the gas sensor array is a fundamental step for improving the chance of the electronic nose to be a good diagnostic tool. The development of further data processing algorithms, based on specific factors connected to the disease analysed, may lead to higher level of selectivity and accuracy of the electronic nose responses.

Pavlou et al. [53] tested the use of an electronic nose as a diagnosis tool for UTI, obtaining good and promising results. They used a specifically tailored conducting polymer sensor array, based on change in electrical resistance, on urine samples, with the aim of investigating the potential ability of the device to discriminate between samples of healthy and sick patients. They carried out two different experiments based on a different sample delivery system and different data processing. In the first experiment, they used an intelligent system based on Genetic Algorithms (a heuristic method used to generate solutions to optimization problems, using techniques inspired by natural evolution), back propagation neural networks (GA-NN) and PCA & DFA-cv (discriminant function analysis and cross validation) sensor parameters. In the second one, they used two genetic training algorithms processing data through a parallel evolutionary succession process towards competent NN solutions. In each test, the prediction rate was very high, proving the high sensitivity of the gas sensor array to urinary tract infections. More in detail, in the first case (i.e., GA-NN) a sensitivity of 100% was achieved, and 98% in the second case (i.e., back propagation NN). The graphical visualization of the dataset was performed by means of PCA and DFA.

Kodogiannis et al. [54] focused their research on the combination of electronic nose and neural networks in order to analyse human urine samples in vitro by the application of an intelligent diagnostic model, develop an extended normalized function (ENRBF) classifier, evaluate its performance, and implement a multiple-classifier scheme for non-linear pattern. They improved the electronic nose performance by the introduction of a new algorithm for data processing. In this way, they were able to prove that the conducting polymer sensor array was suitable for identifying specific bacterial pathogens with accuracy and speed. The parameter accuracy, sensitivity, specificity and predictability obtained was all equal to 100%.

Recently, Roine et al. [55] demonstrated the applicability of a new model of electronic nose to discriminate the most common UTI pathogens from the gaseous headspace of culture plates. More in detail, culture samples containing four most common UTI bacteria (*E. coli*, *S. Saprophyticus*, *E. Faecalis*, *Klebsiella*) and sterile culture plates were analysed using a ChemPro 100i device (Environics Inc., Mikkeli, Finland), consisting of ion mobility spectrometry (IMS) cell and six semiconductor sensors; data were processed with LDA and LR (logistic regression). The electronic nose showed a high performance of sensitivity and specificity, equal to 95% and equal to 97%, respectively, for the discrimination of sterile and infected samples by processing the data with LR. By using LDA, the sensitivity and specificity achieved in the classification of four different bacteria and sterile culture, were equal to 95% and equal to 96%, respectively. The fact that they used a simple and quick sampling method that nevertheless achieved very good results is very interesting: while in previous experiments others used complicated and long sampling methods, researchers here have simply connected the culture plate to the device. They also emphasized that though it took 15 min for the measurement, already after 5 min a significant discrimination between samples is reached. Researchers are committed to improving the measurement cycle in order to further reduce the time, already considerably short for clinical analysis.

Kodogiannis et al. [56] developed an advanced hybrid neural network scheme using two different algorithms and evaluated its performance, studying human urine samples by the application of an electronic nose in order to diagnose UTI. They used a gas sensor array composed of 14 electroconductive polymers chemoresistors. They also employed an innovative approach for data analysis: they decomposed the system into other subsystems, collected data from these ones and then combined the results via a gating network. The principle this work is based on is that every subsystem is “an expert” in some local area of the feature space. They developed four subsystems, each associated with four parameters extracted from the sensor response: absorption (maximum rate of change of resistance), desorption (maximum negative change of resistance), area under the curve and divergence (maximum step response). The issue was how to combine the data collected from the subsystems in a unique response in order to make a decision based on the whole space. For this purpose, two different algorithms were used, the Expectation Maximum (EM) algorithm and a Split and Merge EM (SM) process. The EM process is very dependent on the initial values and this can create problems in computing the sensor response, whereas the SMEM algorithm is nevertheless an improved version of the first one and provided better results at the end of the experiment. The parameters of accuracy, sensitivity, specificity and predictability obtained after combining the features with the above mentioned methods, all turned out to be equal to 100% for the fuzzy integral fusion method. Instead, the adaptive-fuzzy-logic-system (AFLS) present a prediction rate equal to 92.86%. Based on these results they reached the conclusion that the application of a gas sensor array combined with artificial intelligence may lead to rapid and accurate diagnosis.

Sabeel et al. [57] used a Cyranose E-320 electronic nose system to classify different urine VOCs so as to be able to diagnose diabetes and bacterial infections. The array consisted of 32 conducting polymer sensors and data processing was performed by PCA. The researchers analysed the presence of mucus or bacteria, whose abnormal range highlights the existence of a pathological status. The authors declare that the study suggests the possibility of using e-nose as an early detection system for diseases such as diabetes and bacterial infections. Unfortunately, this statement is only based on a qualitative data analysis, since the authors present PCA graphs without providing any further details about accuracy of classification, sensitivity, or specificity. For this reason, the discussion of the obtained results was more vague compared to other studies. In addition the sample preparation details, such as sample storage temperatures are not indicated, and it is not specified if the samples was incubated before analysis, nor are the investigated bacteria types specified, thus making it difficult to provide any critical analysis of this study.

The results presented in the abovementioned studies are summarized in Table 3. The results provided by these experiments prove that urine headspace is an excellent source of information for the detection of the studied bacteria, which are commonly responsible of UTI, and that opportunely trained electronic noses proved able to diagnose UTI without relevant problems. However, as previously discussed in this review, it must be highlighted that the results presented by some of these studies are mainly qualitative and sometimes do not provide sufficient information for a more critical evaluation.

As can be seen from Table 3, the different authors considered more or less the same bacterial species. This is due to the fact that 80% of uncomplicated UTI are caused by *Escherichia coli* and 20% by enteric pathogens such as *Enterococci*, *Klebsiellae*, *Proteus* sp., coagulase-*Staphylococci* and opportunistic fungal pathogens such as *Candida albicans* [75,76].

Also the incubation procedures were approximately the same: about 5 h at 37 °C. The study by Roine [53] represents the only exception, as they analysed the sample directly. This observation may explain the lower performances achieved in this study (i.e., LR used for the discrimination of sterile and infected samples achieved sensitivity of 95% and specificity of 97% LDA used for the classification of four different bacteria and sterile culture plate achieved sensitivity of 95% and specificity of 96%) compared to other works, which declare sensitivities and specificities of 100%. However, such numbers should be taken with due care, as such extremely good results (100% specificity and sensitivity) may sometimes hide other problems connected to correct validation procedures [42,77], which are often not specified in the text. Most studies among those here reported involve the use of conducting polymer sensor arrays (Table 1). It would be interesting to get to know more in detail the nature of the sensors in terms of polymers used in order to be able to evaluate the responses of the electronic noses more critically, but such information is generally not provided. Only Kodogiannis et al. [54,56] report the details of the sensors employed.

Finally, it is worth to discuss an additional study by Persaud et al. [58], which focuses both on UTI and on bacterial vaginosis (BV). Bacterial vaginosis (BV) is the most common cause of vaginitis symptoms among women, whose causes are unknown. However, the condition is characterised by a change or imbalance in the vaginal ecosystem, which is reported to produce biochemical changes in the vaginal fluid that can be analysed by means of an electronic nose.

Persaud et al. [58] used GC-MS combined with an array of conducting polymer (CP) sensor to study its reaction to VOCs due to UTI and bacterial vaginosis. The aim of the experiment was to identify certain compounds in the gases emitted by bacteria in urine samples (UTI) and vagina swabs (BV). Data were processed with PCA, based on two different substances, i.e., ammonia and acetic acid, both present in the urine headspace and correlated to the studied diseases. In both trials, they were able to demonstrate the suitability of using CP gas sensors to analyse urine samples for rapid screening of urinary tract infections, thereby identifying a range of ammonia concentration varying between 8 and 500 ppm, as well as the acetic acid present in vaginal swabs for diagnosis of bacterial vaginosis.

### 2.4. Cancer Diseases

Nowadays prostate cancer (PC) represents along with lung cancer one of the most important male tumours; transitional cell carcinoma (TCC) of the bladder is the most common form of bladder cancer and simultaneously the second most common malignant tumour of the genito-urinary tract.

Recent research proved dogs to be able to detect cancer by sniffing, as described for example in the research by Willis et al. [31] on the diagnosis of bladder cancer by VOCs in urine samples, using dog olfactory system. Based on these observations, some researchers started to analyse PC-specific VOCs for tumour diagnosis [34,35].

Similarly, electronic noses may be able to detect cancer markers from human body fluids, thus having the potential of early stage detection for different types of cancers. The analysis of urine samples for cancer diagnosis is a recent research field: the possibility of the diagnosis of urinary tract cancers in a rapid and accurate way has become of great interest in the last few years.

Bernabei et al. [59] focused their research to develop the electronic nose as an early and non-invasive diagnosis tool for urinary cancers (prostate and bladder). Measurements of urine headspace were performed by means of an electronic nose based on eight quartz crystal microbalance (QCM) gas sensors coated with different metalloporphyrins. They collected samples from patients affected by different urological syndromes, some of them were collected both before and after chirurgical operation, as well as from healthy people as a reference group. Electronic nose data were processed by both principal component analysis (PCA) and discriminant analysis solved by partial least squares (PLS-DA). The results were very good and promising in terms of the discrimination between diseased and healthy people: the evaluation rate reached 100%. There was a partial differentiation between patients affected by either prostate cancer or bladder cancer; furthermore not all the post-surgery samples were identified for the presence of benign prostatic hypertrophy (BPH) that compromise the final results.

Weber et al. [60], in order to provide a more sensitive, rapid and convenient diagnosis system, studied the interaction between the VOCs connected to transitional cell carcinoma (TCC) of bladder and a gas sensor array composed of 12 metal-oxide sensor (MOS), 10 metal-oxide-semiconductor field-effect transistor (MOSFET), a capacitance-based humidity sensor and an infrared-based CO_2_ sensor. Urine samples were collected from patients with urological pathologies (not only with cancer disease) and healthy subjects, and divided into four different groups, each containing every type of sample. Data analysis was performed by PLS-DA. In most cases, an accuracy of 70% was reached, but was lower when regarding patients with other non-cancerous tract infections. More in detail, the experiments carried out on the third group (i.e., patients aged between 24 and 89 with confirmed noncancerous urological disease, with or without urine dipstick abnormalities) and the fourth group (i.e., patients with BC) showed less selectivity because of the major similarity of the samples (i.e., classification accuracy fell to 65% with 60% sensitivity and 67% specificity). The researchers concluded that more sophisticated pattern recognition is needed to improve results.

Another experiment concerning bladder cancer by Horstmann et al. [61] evaluated the potential of an electronic nose system for the detection of this type of cancer. They used MOS chips with three thin oxide layers, studying the change in conductivity due to the interaction with VOCs. Fresh voided urine was collected from 15 patients with the clinical suspicion of primary or recurrent bladder cancer and 21 patients without bladder cancer but benign urological conditions. Data were processed employing PCA and discriminant analysis. During the trial, four false positives were found in patients of whom all four histopathologically had urocystis; considering only the patients with bladder cancer, they were all classified correctly with a specificity of 100%. The results of this pilot study revealed the high potential of the electronic nose in the detection of bladder cancer with an overall sensitivity of 75% and specificity of 86% necessitating further investigations.

D’Amico et al. [62] investigated the possible discrimination between VOCs in urine by means of an electronic nose in order to show the possibility to use it as a prostate tumour diagnostic tool. As a matter of fact, high levels of prostate-specific antigen (PSA) are usually a common symptom of PC, but measuring the amount of that protein is not a reliable diagnosis tool since it entails a high level of uncertainty [34]. Biopsy is more reliable, but also more invasive. In order to get a less invasive diagnosis tool, researchers investigated the VOCs in urine as a possible source of information for detecting prostate cancer. They used eight non-selective gas sensors, coated with metalloporphyrins as detection system, and as data processing system partial-least square discriminant analysis (PLS-DA). The urine of each patient was collected twice, thus executing two different trials. Two control samples were misclassified as diseased after putting together the data relevant to the two different urine samples (relevant to two different urinations). Promising results were achieved, demonstrating that urine headspace is a good source of information for prostate cancer. However, as highlighted by the authors themselves, this pilot study is not sufficient and thus needs to be enlarged in terms of population and the urine sampling procedure must be standardized in order to confirm results. It is also important to observe that in this study the results are reported in a qualitative form by means of PLS-DA, but data about classification accuracy, sensitivity and specificity are not discussed.

Santonico et al. [63] resumed the previous study [62] and analysed urine headspace by a gas sensor array, searching for a VOCs fingerprint of PC. In this case they used eight quartz-microbalance sensors covered with different metalloporphyrins in order to study their change in frequency after interacting with urine headspace. Data were elaborated by means of PLS-DA using the “leave one out” criterion. The samples were collected between healthy subjects as control reference and patients affected with prostate cancer. No particular problems were noticed by the authors during their experiments, and the high match between electronic nose results and biopsy results confirmed the high potential of the adopted electronic nose system. However, also in this case, the presented results are only qualitative. Moreover, another critical aspect is associated with the definition of the control group of healthy people, due to the impossibility to exclude for certain the presence of a tumour even after biopsy [34]. As a matter of fact, even with both negative PSA and biopsy, about 10%–15% of the male population over 50 years statistically is affected by a non-identified tumour.

Asimakopoulos et al. [64] evaluated the efficacy of PC detection by electronic nose based on the analysis of human urine samples collected from patients before prostate biopsy, in order to perform VOCs analysis without knowing the results. The device was equipped with eight non-selective gas sensors, each coated with different metalloporphyrins, and data were processed by PLS-DA. They demonstrated a potential role of electronic nose in identifying healthy patients with negative prostate biopsy, with a high accuracy rate. More in detail, the electronic nose recognized 10 out of the 14 cases of PC, that is four false negatives, and 25 out of the 27 negative samples, that is two false positives. The sensitivity was equal to 71.4% and specificity was equal to 92.6%. These results were obtained by analysing only the first part of urine stream during urination. The authors attribute this result to an increased contents of elements of prostatic secretion. The obtained results suggested researchers other areas to focus future research on electronic nose, such as a system miniaturization in order to develop a small, disposable device, or the identification of specific VOCs in order to improve the interaction between compounds and specific sensors.

Roine et al. [65] evaluated electronic nose performance in discriminating PC from BPH using urine headspace. Urine samples were collected from patients with confirmed prostate cancer and from patients with BPH. The electronic nose used was a commercially available model (ChemPro^®^ 100, Environics Inc., Mikkeli, Finland), equipped with ion mobility cell consisting of 8 electrode strips and a metal oxide based semiconductor cell. Data processing was performed by LDA and leave-one-out cross-validation (LOOCV). In both cases, high sensitivity and specificity rates were achieved, proving that the tested electronic nose is a good diagnostic tool for PC. More in detail, the sensitivity and specificity were respectively equal to 78% and 67% when using LOOCV, instead the sensitivity and specificity were to 82% and 88% when using LDA. In the conclusions of the study the authors highlight that samples were collected only from patients sufficiently symptomatic to require surgical operation and hypothesized that the discrimination rates could be improved when considering patients with mild symptoms. Finally, they suggested to extend the study to the effects produced by other factors (e.g., diet, medications, hydration) on the final results.

Colorectal cancer (CRC) remains one of the leading causes of cancer-related death in Europe and the USA. The challenge is the broad overlapping symptoms exhibited from patients suffering from CRC and from those with other lower gastro-intestinal (GI) diseases, especially very common disorders such as irritable bowel syndrome (IBS). The standard diagnostic test for this disease is colonoscopy with biopsy, an expensive and intrusive diagnostic method. For this reason, there is significant research focus on attempting to find an effective and unobtrusive alternative method for screening at risk individuals. In the scientific literature, there are some research studies regarding the application of electronic nose system on faecal samples as a diagnosis tool for CRC reaching promising results, i.e., proving the instruments to be able to distinguish CRC and adenomas patients from healthy controls with high accuracy and high levels of measurement repeatability [78].

Also urine headspace can be studied as a source of information for CRC markers. Westenbrink et al. [66] used an electronic nose system to distinguish between CRC and IBS patients and healthy controls. They used a Wolf (Warwick Olfaction, University of Warwick, Coventry, UK) system, a particular electronic nose device composed of an array of 13 sensors: eight amperometric electro-chemical sensors, two non-dispersive infrared optical devices and a single photo-ionisation detector. Data were acquired by NI USB-6009 and NI USB-6211 OEM programs and processed with multivariate techniques common to electronic noses, such as LDA. A reasonable separation of all 3 groups was reached (particularly well-clustered groups were observed when distinguishing CRC from IBS), with a sensitivity of 78% and a specificity of 79%, despite of the presence of some inter-group overlapping caused by some outliers. This may be due to the fact that IBS can be considered as a widely-variable collection of symptoms rather than a well-characterised disease state.

According to the results achieved by all the above mentioned experiments, urine headspace and its modification is clearly connected to diseases like urological cancer, and its analysis can give important information about the patient’s clinical conditions. Table 4 reports a schematization of these studies, indicating the electronic nose type used, the considered pathologies, the sample preparation methods, and the numerousness of the studied population. In such clinical studies, this last parameter is of fundamental importance in order to interpret the presented results in terms of reliability and robustness.

In general, the presented results are very encouraging, showing that almost in all cases studied a clear discrimination between sick patients and healthy people was achieved. On the contrary, not always equally significant results were obtained in the discrimination between the VOCs content relevant to various diseases (numerous cases of data overlapping), often due to similarities between the volatile compounds. Based on these evidences, some researchers have highlighted the need for a more in-depth examination not only on the sensor interaction with the VOCs and the data processing system, but also on the study of the VOCs associated with different diseases in order to improve the applicability of the electronic nose. Having a full knowledge of the changes in excreta associated to the studied diseases should lead to a better classification of samples.

Of course, more trials and extensive validation studies are required to improve the results obtained up to now and achieve a higher and more complete level of information, which is necessary in order to make this new technology acceptable as an efficient diagnosis system.

### 2.5. Diabetes

Diabetes is one of the most common chronic diseases caused by metabolic malfunctioning in the carbohydrate metabolism. Diabetes occurs either when the pancreas does not produce enough insulin or when the body cannot effectively use the insulin it produces. Diabetes can be diagnosed by measuring the glucose level in blood. This can be done by means of three methods, all of which are quite complicated and time-consuming. Another method for fast diabetes diagnosis is the measurement of glucose in urine: in general, urine contains very little glucose, people with abnormal high blood sugar often have glucose in their urine because of diabetes. The effect of a high level of sugar in urine can cause a sweet or fruity odour. Therefore, it is possible to think to use an electronic nose for diabetes diagnosis through the direct analysis of urine odour.

Siyang et al. [67] tried to diagnose diabetes by direct measurement of urine odour, connected to high level of glucose in it. They used eight commercial chemical gas sensors, based on change of resistance (TGS sensors). Data were processed by PCA and clustering analysis. Urine samples were collected from people with normal level of glucose in it and then various concentrations of glucose were added in order to simulate the artificial urines of diabetes patients. Fair results were achieved even though the TGS sensor was not designed to detect fruity odour, giving that the employment of a different sensor array could increase the accuracy of the response. Another factor to be considered is the sample temperature. In this case temperature was controlled by a thermostat and varied as follows: 35, 40 and 45 °C. It was observed that the test quality improved by increasing the temperature. As a conclusion, the authors state that their lab-made electronic nose can be a potential device for diabetes diagnosis if applying sample temperature above 45 °C. The study shows graphically how the increase of the temperatures, which increases the vapour pressure of the VOCs and thus their concentration in the sample headspace, produces an improvement of the cluster separation of the urine samples in function of their glucose content, but no further quantitative results are provided.

In the field of diabetes diagnosis, researchers focused also on the analysis of patients’ breath, trying to determine the presence of acetone in the expired breath by means of electronic nose, which is a diabetes symptom. Ping et al. [68] achieved promising results, even if there were some mistakes in the classification of diseased and healthy people.

As a conclusion, it is possible to state that the knowledge about the applicability of the electronic nose for urine samples analysis in the diagnosis of this disease is very lacking and not very encouraging, but it is possible that the use of different and more specific sensors may produce good results also in the next future.

### 2.6. Kidney Diseases

Kidney diseases have increased to an elevated number of cases over the last years. Chronic kidney disease (CKD) is identified by a blood test for creatinine, whose higher levels indicate a decreased capability of the kidneys to excrete waste products. Creatinine levels may be normal in the early stages of CKD, and the condition is discovered if urinalysis (testing of a urine sample) shows the kidney is allowing the loss of protein or red blood cells into the urine. Electronic noses may provide an earlier diagnosis tool for this pathology by analysing the urine headspace seeking blood or other components directly linked to renal disease.

Di Natale et al. [69] studied the electronic nose application in this field, using a quartz-microbalance sensor array and PCA for data processing. Urine samples were collected from healthy children and children affected by kidney diseases presenting blood in urine as a marker. The authors assert that the electronic nose showed good performance in distinguishing samples containing blood from the others. More in detail, a certain correlation between the first three principal components and the indicator of the amount of blood was found. The paper shows a qualitative representation of the results obtained by means of PCA, but no quantitative discussion is provided regarding the electronic nose discrimination capability between healthy children and children affected by kidney diseases presenting blood in urine as a marker. The paper also reports the capability of measuring quantitatively both the pH and the specific weight of the sample respectively with a relative error less than 10% and less than 1%. This measurement was performed using a nonlinear method (i.e., neural networks). This research work was carried out with a reduced number of tests for the calculation of the relative error both in the pH measurement and in the specific weight of the sample. For this reason, in order to increase the statistical meaning of the data set the authors applied the bootstrap resampling procedure.

The research presented was just a first step towards full experimentation with the electronic nose. However, these first results were promising, pointing out the possibility of correlating certain volatile components to the pathology analysed. In the scientific literature there are other studies aiming to identify the presence of kidney diseases such as uremia and renal failure from breathe analysis [74], which aren’t discussed more in detail in this paper since it focuses on the analysis of urine samples.

In conclusion, both urine and breath samples can be considered as a good source of information about kidney diseases, however, more experiments and tests are required with the aim of developing an alternative measurement system for fast and simple clinical analysis.

### 2.7. Bowel Diseases

The fermentation of undigested food in the large bowel, by its resident bacteria, results in the production of several chemicals including volatile gases. This perturbation in gut bacteria is hard to determine and usually requires prolonged culture, often unsuccessful.

Arasaradnam et al. [70] proposed to analyse this components using non-invasive tools, such as an electronic nose and a Field Asymmetric Ion Mobility Spectrometer (FAIMS). FAIMS is a newer technology that separates ionized molecules based on their different mobility in a high electric field. They aimed to demonstrate the ability of both instruments to detect changes in VOCs due to bacterial dysbiosis as a consequence of bowel cleansing. In order to do this, they recruited several patients that underwent different examination: some underwent colonoscopy that requires a standard diet 48 h before and a complete bowl cleansing; some others underwent a flexible sigmoidoscopy, which requires just a partial bowel cleansing 1 h before the procedure. Most of the samples were collected before the examination, but four of them (from patients needing colonoscopy) were collected at specific time points: 48 h before procedure, time of procedure, one week after and two weeks after. The multiplicity of samples guaranteed a complete control of bacteria in a certain lapse of time. They used commercial instruments, i.e., Warwick Electronic Nose, equipped with six metal-oxide sensors, one optical sensor, one pellistor and six electrochemical sensors, and a Lonestar FAIMS instrument (Owlstone Ltd., Cambridge, UK). Data were processed by means of PCA and LDA for electronic nose and Fisher Discriminant Analysis (FDA) for FAIMS. The results obtained turned out to be very promising, as both electronic nose and FAIMS were able to distinguish between those who had complete versus partial bowel cleansing, and also to highlight significant changes in urine VOCs due to a loss of bacterial diversity immediately following bowel cleansing. The authors present their results in a graphical form, showing a good separation of data in tight clusters.

Inflammatory bowel disease (IBD), characterized by chronically recurring abdominal pain or discomfort and altered bowel habits, is one of the most common syndromes observed by gastroenterologists and primary care providers, with a worldwide prevalence of 10% to 15%. IBD is one of several functional gastrointestinal disorders. The quest continues to find simple reliable non-invasive markers to distinguish between Crohn’s disease and ulcerative colitis (UC) and to distinguish between active disease and those in remission. Arasaradnam et al. [71] evaluated the potential use of electronic nose and FAIMS to differentiate between IBD patients from healthy controls and then distinguish between active IBD compared to those in clinical remission. Urine samples were collected from patients with Crohn’s disease, patients with UC and healthy patients as controls. The electronic nose used was a commercial model (Fox 4000, AlphaMOS, Toulouse, France), comprising an array of 18 metal oxide sensors. For FAIMS testing, a commercial setup (Owlstone Lonestar) was deployed. Electronic nose data were processed by PCA and DFA, while FDA was applied to FAIMS data. The analysis showed that differences between the odour profiles were detectable and this could lead to differentiation between sick patients and healthy controls. PCA showed some cases of overlapping due to its nature of non-classified technique, while DFA was able to produce a clearer separation than PCA. Anyway, it was also possible to separate patients between those experiencing a flare and those in remission, thus giving that both electronic nose and FAIMS could be considered efficient diagnostic tools for IBD, as the accuracy achieved in the separation between patients with IBD and control group was above 75%.

Chronic diarrhoea can have many causes. Bile acid diarrhoea (BAD) is one of the commonest and requires expensive imaging to diagnose. More than 98% of bile acids produced are reabsorbed back into circulation, with less than 2% lost in faeces. If this process of reabsorption is perturbed either through disease, surgical removal of a length of bowel or through defects in certain regulatory proteins, then the excess bile that is not reabsorbed spills over into the colon resulting in symptoms of diarrhoea.

Covington et al. [72] tested an electronic nose system and a FAIMS to differentiate between patients with bile acid diarrhoea, ulcerative colitis (a disease with similar symptoms but different causality) and blank controls focusing only on urine samples analysis. The electronic nose system used was equipped with ten different metal-oxide sensors, and data were processed by PCA and LDA. For FAIMS testing, a commercial instrument was deployed and FAIMS measurements were undertaken over a 6 months period; data were processed in a custom LabVIEW program. The data were processed by LDA. Some problems arose with data processing, as results from PCA showed no clear trend and only controls were separated from other samples. By changing the data processing with LDA, better results were obtained and groups appeared clearly separated (the classification success rates was equal to 83%). Based on the test results it was possible to compare the performances of the two different diagnostic tools, giving that FAIMS turned out to be more sensitive and provides a much higher level of information, whereas the electronic nose is simpler, but sensor drift and sensor-to-sensor variation might be a problem.

Disregarding the type of sensor or the technique used, the information gained with these trials are very important, as they established with no doubt that the VOCs/gas signature profiles are different between samples coming from patients with bile acid diarrhoea, ulcerative colitis (type of inflammatory bowel disease) and healthy individuals, giving that their analysis may lead to diagnose the pathology. The changes in gas profiles in patients with BAD compared with those with Ulcerative Colitis and healthy controls confirmed that gut dysbiosis in those with BAD results in a chemical fingerprint that can be detected (altered fermentation profile). VOCs and other vapours are produced as a result of colonic fermentation and, as a consequence, vapours emitted from urine, faeces and breath may include biomarkers of use in the assessment of gastrointestinal disease. Techniques that are able to detect these biomarkers in body fluid samples could become a good replacement for actual expensive diagnosis tools.

### 2.8. Exposure to Toxic Agents

Benzene is a common source of environmental and occupational exposure dangers. It is a component of petroleum products and a trace impurity in industrial products resulting in continued higher occupational exposures in industrial settings, especially in developing countries, occurring through inhalation and/or absorption via the skin. Benzene is metabolized in the human body to phenols, which can be detected in the urine of exposed workers.

Mohamed et al. [73] investigated the use of an electronic nose system to monitor the headspace of biological samples in order to distinguish between exposed and non-exposed Egyptian workers. Blood, urine and exhaled air were collected after an eight-hour working shift, to assure the best results. The electronic nose used for this study was equipped with ten different metal oxide sensors and data were processed by PCA. Blood and urine samples of exposed workers presented higher levels of phenols and muconic acid, two common metabolites of benzene in human subjects linked to benzene exposure; these levels could be easily detected by the electronic nose. However, benzene was not detected in any of the blood samples from both groups or in the urine samples of non-exposed controls. Benzene was detected in the urine samples of the exposed group. The other markers (i.e., phenols and muconic acid) were detected in both fluids for the exposed Egyptian workers. Clear distinction between both groups is evident, meaning the electronic nose was capable of identifying samples from each group with no false-positive (non-exposed controls) or false-negative (benzene-exposed) results. This leads to support the fact that the volatiles found in the air exhaled by workers exposed to benzene (i.e., phenols and muconic acid) are significantly different from those for non-exposed controls.

Thus, the electronic nose may effectively represent a useful tool for the low-cost mass screening and early detection of health dangers associated with the exposure to toxic compounds in the industry.

## 3. Conclusions

In this work we provide an up-to-date review on the potential of the electronic nose as a non-invasive and quick diagnostic tool for the detection of specific diseases associated with the analysis of urine odour. In general, the response provided by the electronic nose is a variation of a certain property of the sensor (e.g., conductivity for MOS sensors, oscillation frequency for QMB) that is transduced into an electrical signal. The overall responses of the sensor array produce a sort of “fingerprint” of the analysed odour as a whole, which gives that the electronic nose isn’t able to identify single chemical compounds. In order to make the instrument sensitive to a wide range of compounds and of their combinations, sensors used in electronic nose systems are typically non-specific, as it is the case for the studies reviewed in this work. For this reasons, the outcomes of these studies do not add information about the chemical nature of the analysed samples, for which other analytical techniques should be used, such as GC-MS, which on the other hand entail the drawback that they are not always suitable in order to provide information about the olfactory properties of a chemical mixture [18]. Based on this functioning principle of the electronic nose, the discrimination between different odours is not operated based on the identification of their chemical composition, but on the differentiation of the odours fingerprints.

This may be considered as a limit of the electronic nose technology, as it does not allow the identification of specific biomarkers associated with the studied diseases. On the other hand, this may be seen as an advantage, since the chemical speciation of a complex fluid with an extremely variable composition such as urine may not necessarily lead to the identification of useful information for disease diagnosis. Moreover, the principle of processing a limited number of non-compound-specific signals thus producing a fingerprint of an odour as a whole is more similar to the odour perception mechanism of the mammalian olfactory system.

However, it is possible to think of a possible future development and use of application-specific “hybrid” systems, consisting of the combination of non-specific sensors together with specific sensors or other devices, capable of both producing a response to the odour as a whole, but also to detect and measure the presence of specific compounds. However, the possibility to apply similar techniques is based on previous achievements in this research field regarding the identification of such specific compounds to be monitored, i.e., of the biomarkers associated with the disease to be diagnosed.

The critical discussion of the current literature in this field was performed by considering each pathology separately, as the application of the electronic nose for the diagnosis of those pathologies showed a different rate of success and highlighted specific limitations and weaknesses related to its use. More in detail, sensors have proven often insensitive to interaction with certain volatile components and data processing models are not always optimized, and have sometimes led to recognition errors. These are some of the reasons that have certainly limited the spread of the electronic nose in the biomedical science, although it certainly has proved as an effective and inexpensive diagnostic tool.

The research is however rather recent and still in progress. There are several aspects that need to be investigated more into depth, not only to develop and improve specific electronic noses for different diseases, but also with the aim to discover and analyse the connections between specific diseases and the body fluids odour. Further research is needed to improve the results obtained up to now; the development of new sensors and data processing methods should lead to greater diagnostic accuracy thus making the electronic nose an effective tool for early detection of different kinds of diseases, ranging from infections to tumours or exposure to toxic agents.

Regarding the data processing methods, based on the information provided in the scientific literature here examined, it is not possible to identify a specific algorithm that clearly prevails compared to others in terms of performances, i.e., classification accuracy, specificity and selectivity. In the discussed research papers, similar results were obtained with very complex algorithms (e.g., neural networks, genetic algorithms) as well as with much simpler methods (e.g., PCA, LDA), giving that up to now it is not possible to give an indication about an absolute optimal data processing procedure. However, it is our opinion that, even though data processing is a crucial aspect to be considered for the successful application of electronic noses in any field, it is much more important to focus the attention on the quality of the data to be processed. This means that further research is needed in order to improve the sensor responses in terms of stability, repeatability, and low noise. Sensor drift is recognized to be one of the main problems associated with the use of gas sensors, and statistics is unable to adjust a bad dataset [42].

Another aspect that in our opinion has not been investigated deep enough up to now, is the sample preparation. In order to produce a good dataset it is not only important to focus on the development of the sensors, but it is also extremely important to optimize the procedures for the optimization of the sample that gets in contact with the sensors. Based on our experience, it is possible to assert that in the next future particular care should be given to the sample preparation procedure, in order to maximize the presence of VOCs of interest in the analysed gas phase, thereby reducing the noise caused by the presence of interfering VOCs. As an example, ammonia typically indicates the beginning of bacterial activity, and if present at high concentrations, it may “cover” the information contained in the signal [79]. This aspect is particularly important when dealing with a biological fluid whose composition is linked to several different environmental and behavioural factors, such as physical activity, diet or exposition to polluting agents.

Another major aspect that should be considered for the popularization of electronic noses as diagnostic tools is the transfer ability of the prediction models, which means that the prediction model trained on one device should be transferable to other devices [80]. This point is particularly important for the large-scale deployment of electronic noses, especially for diagnostic purposes, where sample collection for the instrument training and for the development of the prediction model is typically highly money- and time-consuming [81].

However, thanks to the recent achievements in this field, it is possible to think positive and expect further significant developments, thus believing that electronic noses may soon represent a vital part in monitoring pathologies and diseases epidemiology. In facts, it is possible to think that in the future, medically applied electronic noses might be used not only as diagnosis tools, but, with a specific training, they could possibly be suitable for screening for further testing or for tracking the course of a disease, and maybe even evaluate its degree of offensiveness. This last aspect could be particularly interesting especially in the monitoring of the development of cancer diseases.

As a last consideration it might be worth to highlight that, it is also indubitable that only a multidisciplinary team, that includes clinicians, engineering, biologists, physicians and biochemical scientists collaborate together to understand the complexity of human beings. This way of thinking may further help to clarify concepts, interpret new and old experimental data, indicate alternative experiments and discover even more appropriate diagnostic tools.

## Figures and Tables

**Table 1:** Application of e-nose for evaluation of different diseases using urine samples.

Reference Number	Authors (Year)	Disease Studied	Detection System	Data Processing Methods
[49]	Pavlou et al. (2002)	BC	14 CP	GA, NN, PCA, DFA-cv
[50]	Bruins et al. (2009)	BC	1 MOS	SW-MV, DTW
[51]	Yates et al. (2005)	BC	32 CP (Cyrano Sciences C320, Smiths Detection, Bushey, Hertfordshire, UK)	MPL, ARX, RBFs, non linear ARX
[52]	Aathithan et al. (2001)	BC	4 CP (Osmetech Microbial Analyzer-OMA, Osmetech plc, Crewe, UK)	PCA
[53]	Pavlou et al. (2002)	UTI	14 CP	GA, NN, PCA, DFA-cv
[54]	Kodogiannis et al. (2008)	UTI	14 CP (Bloodhound BH-114, Bloodhound Sensors Ltd., Leeds, UK)	implementation of an advanced NN
[55]	Roine et al. (2014)	UTI	6 MOS (ChemPro 100i, Environics Inc., Mikkeli, Finland)	LDA, LR, PCA
[56]	Kodogiannis et al. (2005)	UTI	32 CP (Cyranose E-320, Sensigent, Baldwin Park, CA, USA )	NN, EM, SM
[57]	Sabeel et al. (2013)	UTI	32 CP (Cyranose E-320, Sensigent, Baldwin Park, CA, USA)	PCA
[58]	Persaud et al. (2005)	UTI	CP	PCA
[59]	Bernabei et al. (2007)	CD	8 QCM	PCA, PLS-DA
[60]	Weber et al. (2011)	CD	12 MOS, 12 MOSFET, 1 capacitance-based humidity sensor and 1 IR-based CO_2_ sensor	PLS-DA
[61]	Horstmann et al.(2015)	CD	MOS	PCA
[62]	D’Amico et al. (2012)	CD	8 QCM	PLS-DA
[63]	Santonico et al. (2014)	CD	8 QCM	PLS-DA
[64]	Asimakopoulos et al. (2014)	CD	8 QCM	PLS-DA
[65]	Roine et al. (2014)	CD	8 electrode strips and 1 MOS (ChemPro^®^ 100, Environics Inc., Mikkeli, Finland)	LDA, LOOCV
[66]	Westenbrink et al. (2014)	CD	8 amperometric electro-chemical sensors (Alphasense Ltd., Great Notley, Essex, UK), 2 non-dispersive IR, optical devices (Clairair Ltd., Witham, UK) and 1 photo-ionisation detector (Mocon, Minneapolis, MN, USA).	LDA
[67]	Satetha Siyang et al. (2012)	D	8 commercial chemical gas sensors, based on change of resistance (TGS sensors)	PCA, CA
[68]	Ping et al. (1997)	D	MOS	NN, fuzzy cluster pattern recognition
[69]	Di Natale et al. (1999)	KD	QMB	PCA
[70]	Arasaradnam et al. (2012)	BD	6 MOS, 1 optical IR sensor, 1 pellistor, 6 electrochemical sensors	PCA, LDA
[71]	Arasaradnam et al. (2013)	BD	18 MOS (Fox 4000, AlphaMOS, Toulouse, France)	PCA
[72]	Covington et al. (2013)	BD	10 MOS	PCA, LDA
[73]	Mohamed et al. (2013)	exposure to toxic agents	10 MOS (PEN3, Airsense Analytics GmbH, Schwerin, Germany)	PCA

Abbreviations: Genetic algorithms (GA), neural networks (NN), principal components analysis (PCA), discriminant function analysis and cross-validation (DFA-cv), Sliding Window-Minimum Variance matching adaptation of the Dynamic Time Warping algorithm (SW-MV, DTW), Multilayer perceptron (MLP), autoregressive exogenous type (ARX), Radial basis functions (RBFs), parametric Discriminant Function Analyses and cross validation (DFA-cv), linear discriminant analysis (LDA), logistic regression (LR), Expectation Maximization algorithm (EM), Split and Merge (SM), partial least squares-discriminant analysis (PLS-DA), leave-one-out cross-validation (LOOCV), cluster analysis (CA), bacteria cultures (BC), urinary tract infections(UTI), cancer diseases (CD), diabetes (D), kidney diseases (KD), bowel diseases (BD), metal oxide semiconductors (MOS), quartz microbalances (QMB or QCM), metal oxide semiconductor field effect transistor (MOSFET), conducting polymer (CP).

**Table 2:** Scheme of the studies regarding the detection/identification of bacteria cultures.

Ref. No.	Main Author	E-Nose Type	Bacterial Species	Culture Broths	Results
[49]	Pavlou et al.	e-nose system with 14 CP	*Clostridium* spp.;	36 blood agar plates (Merck, Anaerobic)	PCA→94%
			*Bacteroides fragilis*		
[50]	Bruins et al.	Mono-nose	*Clostridium difficile*, *Enterobacter cloacae*, *Enterococcus faecalis*, *Escherichia coli*, *Klebsiella* spp., *Proteus mirabilis*, *Pseudomonas aeruginosa*, *Salmonella* spp., *Staphylococcus aureus*	BD-BACTEC™—Plus-Anaerobic/F Medium with the addition of 0.1 mM FeCl_3_	SW-MV, DTW→87%
[51]	Yates et al.	C320	Blood used as the growth medium: various species of bacteria—including methicillin susceptible *Staphylococcus aureus*, methicillin resistant *Staphylococcus aureus*	Blood Urine	*URINE:* - ARX→71% - RBF→65% - non linear ARX→80% - BLOOD: - ARX→94% - RBF→71% - Sammon maps + RBF→100% - nonlinear ARX→72%
			Urine from routine medical screening		
[52]	Aathithan et al.	OMA	*Escherichia coli*, *Klebsiella pneumoniae*, *Proteus mirabilis*, *Staphylococcus aureus*, *Staphylococcus saprophyticus*, *Enterococcus faecalis*	Agar	PCA→sensitivity 84%, specificity 88%

**Table 3:** Scheme of the studies regarding the detection/identification of UTI.

Reference Number	Main Author	E-Nose Type	Bacterial Species	Culture Broths	Incu-Bation	Results
[53]	Pavlou et al.	BH114-Blood-hound	*Escherichia coli*, *Proteus* spp., *Staphylococcus* spp.	Agar culture, brain heart infusion broth and cooked meat broth	4 h 1/2 37 °C	- GA-NN→prediction rate 100%, - back propagation NN→prediction rate 98%
[54]	Kodogiannis et al.	BH114-Blood-hound	*Escherichia coli*, *Proteus* spp., *Staphylococcus* spp.	brain heart infusion broth and bovine serum	5 h 37 °C	fuzzy integral methodology: (Advanced NN)→accuracy 100%, sensitivity 100%, specificity 100%, predictability 100%
[55]	Roine et al.	ChemPro 100i	*Escherichia coli*, *Staphylococcus* *saprophyticus*, *Klebsiella* species, *Enterococcus faecalis*	cysteine lactose electrolyte deficient medium (CLED) Agar	no	- LR used for the discrimination of sterile and infected samples→sensitivity 95%, specificity 97% - LDA used for the classification of four different bacteria and sterile culture plate→sensitivity 95%, specificity of 96%
[56]	Kodogiannis et al.	BH114-Blood-hound	*Escherichia coli*, *Proteus* spp., *coagulase-Staphylococcus* sp.	brain heart infusion broth and bovine serum	5 h 37 °C	- fuzzy integral fusion method (NN)→accuracy 100%,sensitivity 100%, specificity 100%, predictability, 100% - adaptive-fuzzy-logic-system (AFLS) (NN)→prediction rate 92.86%
[57]	Sabeel et al.	Cyranose E-320	-	-	-	-

**Table 4:** Scheme of the studies regarding cancer diseases.

Reference Number	Main Author	E-Nose Type	Patho-Logies	Sample Preparation Method	Results	Sample
[59]	Bernabei et al.	ENQBE	PC, BC	Urines collected in the morning, before any food intake. Stored at 25 °C for the necessary time to obtain a steady headspace. 10 mL of the headspace injected into a 2 L sterile bag pre-filled with N_2_	(1) PLS-DA→ discrimination between the patients and to the healthy controls 100%. (2) PCA→not a complete discrimination between the two tumors, but a sort of gradual differentiation (3) PCA→migration of post-surgery patients towards the healthy class	131: 25 BC, 12 PC, 29 BPH, 33 various urological pathologies, 18 healthy control (19 patients measured twice, before and after the surgical treatment of cancer)
[60]	Weber et al.	NST 3320 Lab Emission Analyser	BC	Storage at −80 °C, defrosted at room temperature (21 °C). samples aliquoted into vials and incubated for 1 h at 38 °C	(1) PLS-DA→healthy volunteers vs. bladder cancer patients: sensitivity 70%, specificity 70% (2) PLS-DA→data of healthy controls suffer from other non-cancerous urological diseases: classification accuracy 65%, sensitivity 60%, specificity 67%	89: 30 patients with BC, 59 healthy control
[61]	Horstmann et al.	e-nose based on a MOS sensor	BC	-	PCA→sensitivity 75%, specificity 86%	36: 15 BC 21 healthy control (with no pathologies or benign urological conditions like BPH and UTI)
[62]	D’Amico	e-nose University of Rome “Tor Vergata”	PC	Samples collected in sterile urine boxes with a dedicated top to extract the headspace of urine for analysis. No more information are reported	PLS-DA→the results reported are only qualitative; data regarding classification accuracy, sensitivity and specificity of the adopted method are not discussed	21
[63]	Santonico et al.	e-nose University of Rome “Tor Vergata”	PC	Measurements performed at room temperature. No more information are reported	PLS-DA→the results reported are only qualitative, no quantitative data are provided about the adopted method	41: 27 healthy control, 14 PC
[64]	Asimakopoulos et al.	e-nose University of Rome “Tor Vergata	PC	Measurement performed within 2 h from the collection	PLS-DA→sensitivity 71.4%, specificity 92.6%	41
[65]	Roine et al.	ChemPro 100-eNose	PC	Sample defrosted and pipetted to a plate heated and maintained at 37 °C	- LOOCV→sensitivity 78%, specificity 67% - LDA→sensitivity 82%, specificity 88%	74: 50 PC; 24 healthy control (15 BPH, 9 patients provided samples 3 months postoperatively)
[66]	Westenbrink et al.	WOLF system	CRC	Storage at −80 °C, defrosted overnight at 5 °C. Samples heated to 40 °C for 5 min	LDA→sensitivity 78%, specificity 79%	92: 39 CRC; 35 IBS; 18 healthy control
